# Genome-wide analysis discloses reversal of the hypoxia-induced changes of gene expression in colon cancer cells by zinc supplementation

**DOI:** 10.18632/oncotarget.395

**Published:** 2011-12-24

**Authors:** Michal Sheffer, Amos J. Simon, Jasmine Jacob-Hirsch, Gideon Rechavi, Eytan Domany, David Givol, Gabriella D’Orazi

**Affiliations:** ^1^ Department of Physics of Complex Systems, Weizmann Institute of Science, Rehovot 76100, Israel; ^2^ Department of Pediatric Hematology-Oncology, Chaim Sheba Medical Center and Sackler School of Medicine, Tel-Aviv University, Tel-Aviv, Israel; ^3^ Department of Molecular Cell Biology, Weizmann Institute of Science, Rehovot 76100, Israel; ^4^ Department of Experimental Oncology, Molecular Oncogenesis Laboratory, National Cancer Institute Regina Elena; Rome, Italy; ^5^ Department of Oral Sciences, Nano and Biotechnology, University “G. d’Annunzio”, Chieti, Italy

**Keywords:** hypoxia, cobalt, pathway analysis, Principal Component Analysis (PCA), cDNA microarray, colon cancer

## Abstract

Hypoxia-inducible factor 1 (HIF-1), the major transcription factor specifically activated during hypoxia, regulates genes involved in critical aspects of cancer biology, including angiogenesis, cell proliferation, glycolysis and invasion. The HIF-1a subunit is stabilized by low oxygen, genetic alteration and cobaltous ions, and its over-expression correlates with drug resistance and increased cancer mortality in various cancer types, therefore representing an important anticancer target. Zinc supplementation has been shown to counteract the hypoxic phenotype in cancer cells, *in vitro* and *in vivo*, hence, understanding the molecular pathways modulated by zinc under hypoxia may provide the basis for reprogramming signalling pathways for anticancer therapy. Here we performed genome-wide analyses of colon cancer cells treated with combinations of cobalt, zinc and anticancer drug and evaluated the effect of zinc on gene expression patterns. Using Principal Component Analysis we found that zinc markedly reverted the cobalt-induced changes of gene expression, with reactivation of the drug-induced transcription of pro-apoptotic genes. We conclude that the hypoxia pathway is a potential therapeutic target addressed by zinc that also influences tumor cell response to anticancer drug.

## INTRODUCTION

Hypoxia is a common state of cancer cells due to the lack of blood supply to the rapidly growing tumor. Hypoxia-inducible factor 1 (HIF-1) is the key factor that mediates adaptive response to hypoxia; it is an heterodimeric transcription factor consisting of the HIF-1b subunit, constitutively expressed in cells, and the HIF-1a subunit whose stability is enhanced by low intracellular oxygen and also by genetic alteration [1]. In normoxia, HIF-1a is hydroxylated by prolyl hydroxylases (PHD1-3) at key proline residues in the oxygen-dependent degradation domain (ODD) facilitating interaction with the E3 ligase, Von Hippel-Lindau protein (VHL), which drives HIF-1a ubiquitination and degradation [2, 3]. Under hypoxic conditions, prolyl hydroxylation is inhibited, thereby stabilizing HIF-1a and EPAS1 (also known as HIF-2α), which can then translocate to the nucleus and bind to constitutively expressed HIF-1b forming the active HIF-1 complex [4]. In a screen from the National Toxicology Program [5], three out of 1408 tested compounds (o-phenantroline, IodoChlorohydroxiquinone and cobalt sulfate) were selected as hypoxia-mimetic whereas cobalt was the only one that interacted with HIF-1 in a manner similar to hypoxia. Thus, cobaltous ions have been shown to inhibit hydroxylation of HIF-1a and therefore induce elevated HIF-1a protein levels, mimicking hypoxia [1]. The interest in HIF-1 comes form the fact that HIF-1 controls the expression of numerous genes involved in many aspects of cancer progression, including angiogenesis (e.g., vascular endothelial growth factor - VEGF), metabolic adaptation (e.g., Glut1), apoptosis resistance (e.g., Bcl2), invasion and metastasis (e.g., c-Met) [6]. The levels of HIF-1a subunit are often increased in most solid human tumors including colon, brain, breast, gastric, lung, skin, ovarian, prostate, renal, and pancreatic carcinoma, rendering tumor cells resistant to conventional chemotherapy and selecting a more malignant and invasive phenotype leading to poor prognosis [6]. Inhibition of HIF-1a may, therefore, represent an attractive strategy with potential for synergism with other antitumor therapies [7, 8].

We found that zinc supplementation to highly invasive and angiogenic glioblastoma cells or to prostate cancer cells, either constitutively hypoxic or after induction of hypoxia, downregulates HIF-1a protein levels and inhibits HIF-1 activity, resulting in the inhibition of VEGF expression, angiogenesis and tumor cell invasiveness [9]. This is relevant in light of recent findings showing that treatment of cancer by anti-angiogenic agents like anti VEGF or small molecule inhibitors of tyrosine kinase may result in hypoxia that selects for more malignant metastatic and invasive cells, that eventually lead the tumors to relapse as a more invasive and metastatic disease [10, 11]. Similar results may apply also for the treatment with antibodies when the signaling triggered by the antibody affects the HIF-1 pathway [12]. Zinc is a trace element that is essential for the normal function of cells and is a cofactor for a wide range of structural proteins, enzymes, and transcription factors involved in key cellular functions such as the response to oxidative stress, DNA damage repair, cell cycle progression and apoptosis [13]. The mechanisms through which gene expression is regulated by zinc are central to cellular homeostasis, thus, zinc is an essential prerequisite for the execution of many signaling pathways in eukaryotes [14]. Preclinical studies have shown that zinc exerts a positive beneficial effect against chemically induced pre-neoplastic progression in rats and provides an effective dietary chemopreventive approach to disease [15]. On the other hand, zinc deficiency has been associated with induction of cancer [16]. Furthermore, *in vitro* and *in vivo* studies by our group have shown that zinc supplementation to cancer cells improves the chemotherapeutic response with reactivation, for instance, of inactive onco-suppressor p53 and apoptosis [17, 18]. Understanding the molecular pathways modulated by zinc under hypoxia may provide the basis for reprogramming signalling pathways for anticancer therapy and hopefully improve classical anticancer therapies. To this aim, we performed a genome-wide expression analysis in colon cancer cells treated with different combinations of hypoxia-mimetic cobalt, zinc and anticancer drug. Our strategy identified differences in gene expression among the combination treatments. The most remarkable result was that zinc reversed gene expression of most genes modulated by hypoxia, including genes involved in metabolism, proteasomal build-up, and amino acid biosynthesis. As a result of hypoxic phenotype reversion, zinc supplementation restored the drug-induced apoptosis, inhibited by hypoxia. Our studies suggest that zinc supplementation to cancer cells may have an effective anticancer outcome by targeting the hypoxia pathway and therefore provide the molecular basis for the combination treatment of tumors by zinc with classical anti-tumoral drugs.

## RESULTS

### Expression of the modulated genes shared between cobalt and hypoxia

Low oxygen as well as cobaltous ions inhibit hydroxylation of HIF-1a and therefore induce elevated HIF-1a protein levels, mimicking hypoxia [1]. Here, we first attempted to evaluate the extent of similarity in gene expression between cobalt and hypoxia treatment by constructing a list of hypoxia genes using hypoxia related gene sets that were published on the MSigDB database [19 by ten different studies [20-29]. This resulted in 150 up-regulated (hypoxia up) and 76 down-regulated (hypoxia down) genes that appeared in at least two out of the ten hypoxia studies (data not shown). When the 150 and 76 modulated genes were intersected with the modulated genes in the cobalt (C) treatment of RKO cells (Supplementary Table S1), the resulting shared genes were found to be 54 out of the 150 ‘hypoxia up', and 12 out of the 76 ‘hypoxia down' genes (Table 1, column C-0). This significant overlap is in agreement with many studies on hypoxia-like effect by cobalt, showing high level of similarity in modulated genes between hypoxia and cobalt treatment [30, 31].

**Table 1:** Hypoxia and cobalt treatment shared genes and their reversal by zinc supplementation The table lists 54 up-regulated and 12 down-regulated genes that are shared between the cobalt treatment (C) of RKO cells and the hypoxia modulated genes reported by at least two out of ten studies taken from the MSigDB [19]. The fold change of gene expression in RKO treated cells is shown for treatment by cobalt compared with untreated cells (C-0) and by treatment with zinc and cobalt compared with cobalt treated cells (ZC-C); negative sign of the fold changes indicates decrease in expression.

Symbol	Gene Title	C-0	ZC-C	References
Up-regulated		Fold change	Fold change	
P4HA1	prolyl 4-hydroxylase, alpha polypeptide I	3.12	−1.32	20-26, 29
ADM	adrenomedullin	6.67	−4.43	20-22, 25-27
ANGPTL4	angiopoietin-like 4	2.83	−1.99	20, 21, 23, 25, 26, 29
BNIP3L	BCL2/adenovirus E1B 19kDa interacting protein 3-like	4.51	−2.09	20-23, 25, 26
NDRG1	N-myc downstream regulated 1	9.40	−5.30	20,21,23,25,26,29
SLC2A1	solute carrier family 2 (facilitated glucose transporter), member 1	1.99	−1.12	20-24, 29
AK3L1	adenylate kinase 3-like 1	1.75	−1.29	21, 23-25, 29
BHLHE40	basic helix-loop-helix family, member e40	4.63	−2.26	20, 21, 24-26
C7orf68	chromosome 7 open reading frame 68	3.00	−2.25	20, 21, 23, 26, 29
CCNG2	cyclin G2	2.01	−2.30	20-22, 24, 25
ENO2	enolase 2 (gamma, neuronal)	3.90	−2.34	20, 21, 23, 25, 26
KDM3A	lysine (K)-specific demethylase 3A	3.98	−1.88	20, 21, 24, 25, 26
P4HA2	prolyl 4-hydroxylase, alpha polypeptide II	1.82	−1.55	20, 21, 24, 25, 26
PGK1	phosphoglycerate kinase 1	1.54	−1.13	20-22, 26, 29
CA9	carbonic anhydrase IX	7.60	−1.35	20-22, 29
EGLN1	egl nine homolog 1 (C. elegans)	2.90	−1.75	20, 21, 25, 26
FAM162A	family with sequence similarity 162, member A	2.35	−1.56	20, 21, 23, 26
GBE1	glucan (1,4-alpha-), branching enzyme 1	3.17	−1.84	20, 21, 25, 26
HK2	hexokinase 2	1.85	−1.17	20-22, 26
PFKFB4	6-phosphofructo-2-kinase/fructose-2,6-biphosphatase 4	16.27	−4.59	20, 24, 26, 29
ALDOA	aldolase A, fructose-bisphosphate	1.74	−1.32	22,24, 29
ALDOC	aldolase C, fructose-bisphosphate	6.93	−3.57	21,23,26
ANG	angiogenin, ribonuclease, RNase A family, 5	1.61	−1.79	21,26,27
ANKRD37	ankyrin repeat domain 37	5.43	−3.73	20,26,29
HMOX1	heme oxygenase (decycling) 1	6.47	−2.87	22, 23, 28
INSIG2	insulin induced gene 2	2.57	−2.11	20, 21, 26
MAFF	v-maf musculoaponeurotic fibrosarcoma oncogene homolog F (avian)	1.87	−1.48	21, 25, 26
PDK1	pyruvate dehydrogenase kinase, isozyme 1	2.56	−1.18	20, 21, 26
PFKFB3	6-phosphofructo-2-kinase/fructose-2,6-biphosphatase 3	1.55	−1.41	20, 21, 26
PGM1	phosphoglucomutase 1	1.97	−1.41	21, 23, 25
SPAG4	sperm associated antigen 4	2.88	−1.19	21, 25, 26
TMEM45A	transmembrane protein 45A	5.45	−2.53	20, 21, 25
ZNF292	zinc finger protein 292	2.69	−1.56	20, 21, 25
ABCB6	ATP-binding cassette, sub-family B (MDR/TAP), member 6	2.73	−1.90	20, 26
ANKZF1	ankyrin repeat and zinc finger domain containing 1	2.74	−1.75	20, 21
CITED2	Cbp/p300-interacting transactivator, with Glu/Asp-rich carboxy-terminal domain, 2	1.71	−1.87	21, 23
CSRP2	cysteine and glycine-rich protein 2	2.10	−2.05	20, 21
FOS	FBJ murine osteosarcoma viral oncogene homolog	2.99	−1.83	21, 22
GYS1	glycogen synthase 1 (muscle)	1.62	−1.27	20, 21
HCFC1R1	host cell factor C1 regulator 1 (XPO1 dependent)	1.69	−1.87	21, 26
KDM4B	lysine (K)-specific demethylase 4B	2.21	−1.82	21, 26
LIMCH1	LIM and calponin homology domains 1	1.99	−1.46	21, 25
RAB20	RAB20, member RAS oncogene family	3.88	−2.20	20, 23
RBPJ	recombination signal binding protein for immunoglobulin kappa J region	1.58	−1.36	21, 26
RIOK3	RIO kinase 3 (yeast)	1.61	−1.32	20, 26
RORA	RAR-related orphan receptor A	3.98	−2.79	20, 26
SAP30	Sin3A-associated protein, 30kDa	1.77	−1.72	20, 24
SCD	stearoyl-CoA desaturase (delta-9-desaturase)	1.72	−1.67	23, 26
SERTAD2	SERTA domain containing 2	1.66	−1.57	20, 23
SLC16A3	solute carrier family 16, member 3 (monocarboxylic acid transporter 4)	1.57	−1.27	24, 28
STBD1	starch binding domain 1	1.58	−1.75	21, 23
WSB1	WD repeat and SOCS box-containing 1	1.79	−1.29	20, 21
YEATS2	YEATS domain containing 2	1.58	−1.26	20, 21
SPOCK1	sparc/osteonectin, cwcv and kazal-like domains proteoglycan (testican) 1	1.57	−1.37	21, 25
**Down-regulated**				
EEF1E1	eukaryotic translation elongation factor 1 epsilon 1	−1.68	−1.01	20, 21, 25
BOP1	block of proliferation 1	−1.68	1.46	21, 25
DDX21	DEAD (Asp-Glu-Ala-Asp) box polypeptide 21	−1.61	1.56	20, 25
IL18R1	interleukin 18 receptor 1	−1.95	1.97	20, 28
NIP7	nuclear import 7 homolog (S. cerevisiae)	−1.58	1.24	20, 21
RANGAP1	Ran GTPase activating protein 1	−1.55	1.29	20, 21
RRP15	ribosomal RNA processing 15 homolog (S. cerevisiae)	−1.65	1.36	21, 25
RRS1	RRS1 ribosome biogenesis regulator homolog (S. cerevisiae)	−1.59	1.56	21, 25
RUVBL1	RuvB-like 1 (E. coli)	−1.80	1.53	20, 25
SLC25A15	solute carrier family 25 (mitochondrial carrier; ornithine transporter) member 15	−1.75	1.33	20, 25
SRM	spermidine synthase	−1.55	1.48	21, 25
WDR4	WD repeat domain 4	−1.51	1.65	20, 25

Among the shared ‘hypoxia up' genes we identified genes involved in carbohydrates metabolism, fructose, mannose, and glycolysis, (i.e., SLC2A1, also known as GLUT1, PGM1, ALDOA, ALDOC, PFKFB3, PFKFB4, GYS1, GBE1, HK2, ENO2 and PGK1), genes involved in oxidoreductase activity (i.e., SCD, P4HA2, P4HA1, HMOX1 and EGLN1), in autophagy and tumor cell survival (i.e., BNIP3L) [32], in pH regulation (i.e., CA9) [33] in multidrug resistance (i.e., ABCB6) [34] in cell survival and proliferation (i.e., ADM, cyclin G2), in angiogenesis (i.e., EGLN1, ANG and ANGPTL4). We also found newly identified HIF-1a target genes such as TMEM45A, ANKRD37 and WSB1 [35], the latter one being involved in ubiquitination and degradation of HIPK2 [36], a putative tumor suppressor and p53 apoptotic regulator [37] that is down-regulated in hypoxia [38], supporting the hypoxia-mimetic function of cobalt.

Since we recently showed that the hypoxic phenotype can be inhibited by zinc supplementation to cancer cells [9, 17], we next evaluated the effect of zinc treatment on the cobalt modulated genes. Interestingly, we found that zinc markedly reverted the differential expression of genes shared between hypoxia and cobalt (Table 1, column ZC-C), in support of our biological results [9, 17]. Although some of the up- and down-regulated genes were reversed by less than 1.5 fold change, the expression levels of most of the ‘hypoxia up' genes (34 out of 54) and 5 of the 12 ‘hypoxia down' genes were reversed by zinc supplementation to cobalt treatment by more than 1.5 fold change (Table 1).

### Zinc supplementation reverses the gene expression pattern induced by cobalt

**Figure 1:**
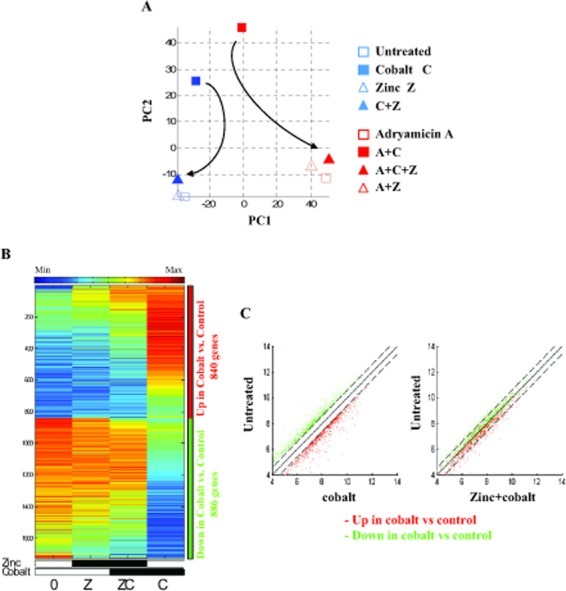
Expression of genes regulated by cobalt and reversed by zinc (A). PCA of the samples, after log2 transformation and averaging between replicates, in the space of all probesets. Filled markers represent cobalt, red markers represent ADR and the triangular markers represent zinc. The arrows indicate the reverse effect of the zinc treatment when applied on cobalt, returning the expression to the original (untreated) position. (B) Heatmap of the genes that were differentially expressed (passed 5% FDR, and had at least 1.5 fold change) between cobalt treated cells (C) and untreated cells (0). Data is centered and normalized after log2 and averaging of replicates, sorted using SPIN [51]. The red sidebar represents up-regulated genes and the blue sidebar represents down-regulated genes. (C) Left panel. Scatter plots of the genes that were up regulated (in red) and down regulated (in green) by cobalt (C), compared with untreated cells (0). Dashed lines represent represent 1.5 fold change (0.585 in log2 scale). Right panel. The same genes when comparing zinc+cobalt (ZC) to untreated cells (0), showing the reversal of gene expression by supplemental zinc on cobalt treated cells.

We next compared global gene expression variation between samples treated with different combination of cobalt (C), zinc (Z) and ADR (A), as shown in Table 2. The number of genes modulated by each treatment is shown in Supplementary Table S1. We used Principal Component Analysis (PCA), a method that reveals the internal structure of high dimensional data in a way which best explains the variance in the data [39]. PC1, the first principal component, shows that ADR treatment had the strongest effect on the cells (Fig. 1A), as PC1 separates the samples into two groups according to the ADR effect, differentiating between samples treated with ADR (red) and without ADR (blue). On the other hand, PC2 separates the cobalt and ADR+cobalt samples (filled red and blue squares) from the rest of the samples (Fig. 1A). Interestingly, zinc treatment shifted the cobalt sample to the untreated and zinc-treated samples (Fig. 1A, see arrow) and the ADR+cobalt sample to the ADR and ADR+zinc samples (Fig. 1A, see arrow), suggesting that zinc counteracts and reverses the effect of cobalt on gene expression. Indeed, this reversal effect was also evident in heatmaps of the normalized expression data, showing that most genes up-regulated by cobalt, marked by the red sidebar, were down-regulated after zinc treatment, while the group of genes down-regulated by cobalt, marked by the green sidebar, was up-regulated after zinc treatment (Figure 1B, compare C with ZC column). This expression reversal was further analysed in the scatter plots showing the same red and green groups of genes comparing cobalt treatment with the untreated sample (Fig. 1C, left panel). The right scatter plot in Fig. 1C shows the same groups when comparing cobalt+zinc with the untreated sample. In this comparison, almost all the genes are within the 1.5 fold change range (marked by dashed lines), meaning that their expression levels were shifted towards their original (untreated) values.

**Table 2:** Schematic representation of the several combination treatments between ADR, cobalt and zinc performed for microarray analysis Each sample was represented by duplicates.

Samples	Abbreviation	ADR	Zinc	Cobalt
**Untreated**	(0)	-	-	-
**ADR**	(A)	+	-	-
**Zinc**	(Z)	-	+	-
**Cobalt**	(C)	-	-	+
**ADR+ Zinc**	(AZ)	+	+	-
**ADR+ Cobalt**	(AC)	+	-	+
**Zinc+ Cobalt**	(ZC)	-	+	+
**ADR+ Zinc+ Cobalt**	(AZC)	+	+	+

**Figure 2:**
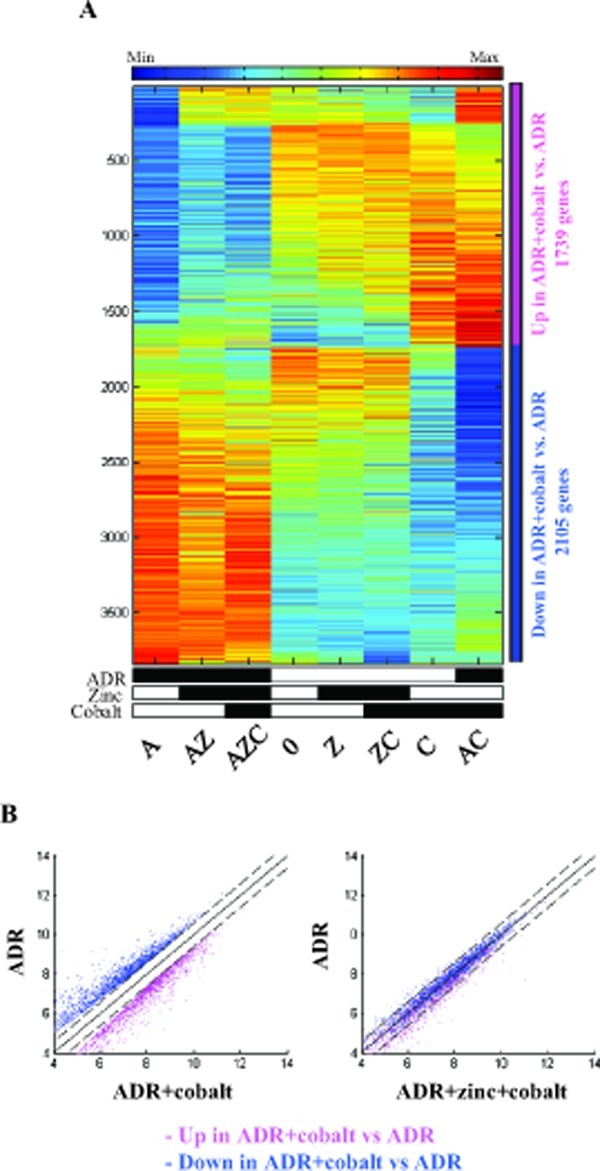
Expression of genes regulated by ADR+cobalt and reversed by zinc (A) Heatmap of the genes that were differentially expressed (passed 5% FDR, and had at least 1.5 fold change) between ADR+cobalt treated cells (AC) and ADR treated cells (A). Data is centered and normalized after log2 and averaging of replicates, sorted using SPIN. The pink sidebar represents up-regulated genes and the blue sidebar represents down-regulated genes. (B) Left panel. Scatter plots of the genes that were up regulated (in pink) and down regulated (in blue) by ADR+cobalt (AC), compared with ADR treated cells (A). Dashed lines represent 1.5 fold changes (0.585 in log_2_ scale). Right panel. The same genes in the comparison of ADR+zinc+cobalt (AZC) to ADR treated cells (A), showing the counter effect of zinc on cobalt treated cells.

The strong effect of zinc on cobalt-induced gene expression was evident in the samples treated with ADR. The heatmap shows that cobalt treatment inhibited the effect of ADR on gene expression (Fig. 2A, compare column A with AC). Interestingly, zinc treatment counteracted the effect of cobalt (Fig. 2A, compare column AC with AZC), restoring the ADR-induced gene expression (Fig. 2A, compare column AZC with A). Of note, zinc treatment per se did not change the ADR-induced gene expression (Fig. 2A, compare column A with AZ), suggesting that the main variation obtained was by cobalt treatment. This expression reversal was also apparent in the scatter plots showing the comparison of AC with A (Fig. 2B, left panel), and then reversal of expression changes in AZC compared with A (Fig. 2B, right panel), showing that zinc supplementation to AC antagonized the effect of cobalt and decreased expression of the up-regulated genes towards their expression levels in A cells. The same applies to the down regulated genes. Altogether, these findings demonstrate that most of the genes that were significantly modulated by cobalt, when compared with the untreated cells, were reversed by zinc almost to their previous expression.

### Zinc restores the ADR-induced pro-apoptotic pathway in the presence of cobalt

**Figure 3:**
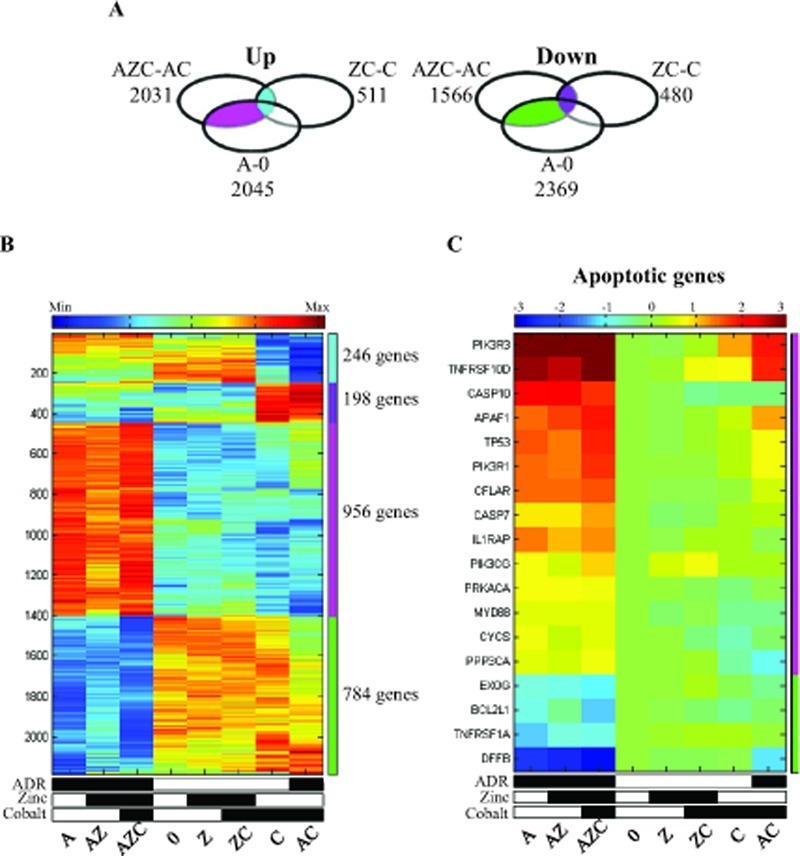
Zinc and adriamycin regulated genes in cobalt treated cells (A). Venn diagram of the up- and down-regulated genes (passed 5% FDR, and had at least 1.5 fold change), following zinc supplement on cobalt treated (ZC) compared with cobalt treated cells (C), zinc supplement to ADR+cobalt treated cells (AZC) compared with ADR+cobalt treated cells (AC), and when comparing ADR treated cells (A) with untreated cells (0). The light blue and violet groups represent genes that are shared between AZC-AC and ZC-C (246 up- regulated and 198 down-regulated genes, respectively). The pink and green groups represent genes that are shared between AZC-AC and A-0, not including ZC-C (956 up-regulated and 784 down-regulated genes, respectively). The intersection of all three comparisons (i.e. AZC-AC and ZC-C and A-0) contains 28 up regulated and 31 down regulated genes. (B). Heatmap of the genes that belong to the colored groups (see Venn diagram, sidebar). Expression values are centered and normalized after log2 and averaging of replicates, sorted using SPIN. (C). Heatmap of apoptotic genes that were regulated by zinc addition to ADR+cobat treated cells. Expression values are presented as log2 fold change compared with the untreated cells. The differences between AZC column and AC column are at least 1.5 fold changes. The up-regulated genes belong to the pink group and the down-regulated genes belong to the green group.

The next step in our analysis was the identification of the cellular pathways that are enriched within groups of modulated genes by the different treatments (see Methods). We first analysed the set of genes modulated in the comparison of AZC with AC treatments (denoted by AZC-AC in Supplementary Table S1). The result is shown in the Venn diagrams and includes 956 up-regulated genes (Fig. 3A, left panel, pink) and 784 down regulated genes (Fig. 3A, right panel, green). Most likely, the genes of these groups, which intersected with genes modulated in the A-0 comparison but not with ZC-C comparison, are good candidates to include genes for apoptotic response. Another group of genes that are modulated by zinc either in the presence or in the absence of ADR, is represented by the intersection of AZC-AC with ZC-C, and may not contribute to the chemosensitivity of the cells, since cells treated with zinc+cobalt do not undergo apoptosis [17]. This intersection includes 246 up-regulated genes (Fig. 3A, left panel, light blue) and 198 down-regulated genes (Fig. 3A, right panel, violet). The normalized expression levels of the genes that belong to these two groups are shown in the heatmap using the same colors than the Venn diagram, indicated on the side bar (Fig. 3B). The first two clusters of genes (light blue, violet) showed differential expression when comparing their expression in the cobalt treated samples (AC, C) versus the other samples. The third and fourth clusters (pink, green) showed differential expression when comparing the ADR related samples (A, AZ, AZC) with the rest of the samples. The reversal by zinc supplementation to cobalt treated cells in the pink and green clusters occurred only in the presence of ADR (Fig. 3B, AZC vs. AC). In order to determine putatively affected pathways, enrichment analysis was performed on these clusters (see Methods). The first enrichment analysis was performed on genes belonging to the pink and green clusters (Fig. 3A, 3B). We found that the apoptotic pathway showed one of the highest score for enrichment, comprising 18 genes between up-regulated pro-apoptotic genes (i.e., caspase 7 and 10, cytochrome C (CYCS) and the apoptotic protease-activating factor APAF1, etc) and down-regulated anti-apoptotic genes (i.e., inhibitor of cell death BCL2L1, etc.) (Table 3). The heatmaps of the normalized expression data show that the apoptotic genes, up-regulated by ADR (A), were downregulated by cobalt (AC) but restored by zinc (AZC) (Fig. 3C). As expected, the apoptotic genes were neither up-regulated by zinc nor in zinc-cobalt treatment (Fig. 3C, ZC).

**Table 3:** Pathways that were found to be enriched with genes modulated by zinc in AZC-AC and A-0 Enrichment analysis of the cluster genes shown in the heathmaps in Fig. 3A (pink and green groups) (see Methods). The arrows indicate the up- and down-regulated genes. Numbers in parenthesis indicate the number of modulated gene.

AZC-AC AND A-0
Chromosome 6p22 (22), Histones (16) [↑] HIST1H2BO, HIST1H3H, LOC222699, PEX6, SOX4 [↓] DTNBP1, HIST1H1E, HIST1H2A (G, H, L), HIST1H2B (C, D, F, I, J, N, I), HIST1H3 (B, F), HIST1H4B, NUP153, ZNF322A RNA polymerase I, III and mitochondrial transcription (28, including the Histones) [↑] KAT2B, POU2F1, PTRF [↓] EHMT2, GTF2H2, GTF3C2, H2AFX, NFIC, POLR1A, POLR3E, POLRMT, SNAPC5 Packaging of telomer ends (13, including 11 Histones) [↓] H2AFX, POT1 Transcription from RNA polymerase II (58) [↑] ARID4A, ATF5, CHD4, CITED (1, 2), ELL2, FOS, FOSB, GTF2B, HEXIM1, HOXC5, IRF (1, 3, 7, 9), KLF (5, 12), LITAF, MEIS2, MTF1, NR6A1, PLAGL1, SKIL, SOD2, STAT (1, 2), SUPT4H1, TBX3, TCF7, TCF7L2, TFAP2A, TP53, TRIM24, ZNF367 [↓] ABT1, ATF4, E2F6, FOSL1, FUBP1, GMEB2, MDM4, MED20, MED7, MYC, NFIC, RNF14, SMARCB1, SNAPC5, SRCAP, SREBF1, TCERG1, TFAP4, TGFB1I1, TNFRSF1A, TRIP4, VPS72, ZNF136, ZNF202
Apoptosis (18) [↑] APAF1, CASP10, CASP7, CFLAR, CYCS, IL1RAP, MYD88, PIK3CG, PIK3R1, PIK3R3, PPP3CA, PRKACA, TNFRSF10D, TP53 [↓] BCL2L1, DFFB, EXOG, TNFRSF1A
Colorectal cancer (14) [↑] CYCS, FOS, PIK3CG, PIK3R1, PIK3R3, RALGDS, TCF7, TCF7L2, TGFB3, TGFBR1, TP53 [↓] ARAF, JUN, MYC
chr9q22 (16) [↑] C9orf130, CTSL2, HABP4, NINJ1, NR4A3, PTCH1, PTPDC1, RMI1, TBC1D2, TDRD7, TGFBR1, ZNF367 [↓] C9orf21, NOL8, RNF20, XPA

### Enrichment analyses of the cluster genes showing additional modified pathways

In addition to the apoptosis pathway, Table 3 reflects the effect of ADR on chromatin and the clusters that contain these genes showed enrichment for genes involved in chromatin structure like histones, some of them involved with enzymes of ribosomal RNA synthesis like RNA polymerase I, and III. Many (over 50) of the genes in this group are involved in facilitating transcription of coding RNA by RNA polymerase II. In addition, genes in these clusters take part in different signaling pathways such as insulin receptor, PI3K, TP53, TGFB that are also related to colorectal cancer. The pathways enriched within the light blue and pink clusters (Fig. 3B) reflect the effect of zinc on cobalt treatment and are mainly involved in carbohydrates metabolism, in protein synthesis and in regulation of metallothioneins (Table 4). Protein translation is one of the processes down-regulated in hypoxia [40]. In this regard, we found increased activation by zinc of pathways, such as metabolism of amino acid, aminoacyl tRNA biosynthesis and transporters of amino acids that will result in increase of protein synthesis (Table 4). On the other hand, the genes that were down-regulated by zinc are enriched in pathways of protein degradation and in proteolytic enzymes associated with protein degradation (Table 4). Decrease in the expression of these genes results in decrease of protein degradation in the cells, which complements the increase in protein synthesis.

**Table 4:** Pathways that were found to be enriched with genes modulated by zinc in both AZC-AC and ZC-C Enrichment analysis of the cluster genes shown in the heathmaps in Fig. 3A (light blue and violet groups) (see Methods). The arrows indicate the up- and down-regulated genes. Numbers in parenthesis indicate the number of modulated gene.

ZC-C AND AZC-AC
Proteasome complex (13) [↓] PSMA2, PSMB (3,5), PSMC (1,4, 6), PSMD (1, 3, 4, 6, 11, 12), SHFM1 Protein catabolic process (6) [↓] MDM2, NPLOC4, UBE4B, UBR3, UFD1L, VCP
Amino Acid transporters and metabolism (11) [↑] SLC1A (4, 5), SLC7A (1, 5, 11), SLC38A1, SLC43A1, ASNS, CPS1, GOT1, PYCR1 Aminoacyl tRNA biosynthesis (5) [↑] AARS, CARS, MARS, MARS2, WARS Other SLC mediated transmembrane transport (5) [↑] SLC2A10, SLC31A1, SLC4A7, SLC6A9, SLC9A2 Glycine, serine and threonine metabolism (5) [↑] CTH, PHGDH, PSAT1, PSPH [↓] ALAS1
Steroid metabolism (6) [↑] TM7SF2 [↓] HMGCS1, HSD17B4, IDI1, MVD, SC4MOL
Gluconeogenesis (5) [↑] GOT1, PCK2 [↓] ALDOC, ENO2, PFKFB4
Protein folding (6) [↑] ARL2 [↓] DNAJB2, LMAN1, PFDN4, TBCE, TTC1
chr16q13 (5), Methallotionines (4) [↑] MT1E, MT1F, MT1JP, MT1X [↓] POLR2C Other Methallotionines (2) [↑] MT1G, MT1M

## DISCUSSION

Hypoxia is a common state within many growing tumors because of lack of adequate blood supply, that promotes cell survival and blocks cell death. Therefore, targeting hypoxia is an attractive strategy to inhibit tumor growth and reactivate drug response. In the present work we aimed at evaluating the global gene expression pattern of colon cancer cell line, treated with the hypoxia-mimetic cobalt and the antagonistic effect of zinc on the gene expression level and particularly with regard to response to drug (ADR) treatment. As demonstrated here, zinc reverted almost completely the hypoxia-induced gene expression. This was markedly evident by using the Principal Component Analysis (PCA) [39], a method that reveals the internal structure of high dimensional data in a way which best explains the variance in the data. This method, clearly substantiated how the zinc treatment shifted the cobalt samples near the untreated or zinc-treated samples (see Fig. 1A, 1B). The positive effect of cobalt inhibition was then corroborated with drug treatment (see Fig. 1A, Fig. 2).

How could zinc down-regulate the hypoxia-induced gene expression? One mechanism could be through inhibition of HIF-1 activity that mediates adaptive responses to changes in tissue oxygenation. Thus, our previous findings showed that zinc supplementation to cancer cells downregulates HIF1α/2α protein levels with a mechanism that involves PHD and VHL, leading to inhibition of HIF-1 activity [9]. This was particularly interesting *in vivo*, where the zinc-inhibitory of HIF-1a was reached inside the xenograft tumor injected in nude mice, meaning that oral zinc administration is able to reach the tumor site and modify the intratumoral HIF-1a expression [9]. The specific effect of zinc on HIF-1 activity was evident here at the molecular level, where the list of genes up-regulated by cobalt treatment and then reversed to their initial expression values by zinc supplementation (see Table 2), largely reflects the HIF-1 target genes (i.e., SLC2A1, BNIP3L, CA9, HK2, ALDOC, TMEM45A, WSB1, etc.) [6, 35]. A large overlap with hypoxia related genes compiled by MSigDB database [19] on the basis of ten different studies [20-29] supported the hypoxia-like effect of cobalt.

Is it possible to clinically exploit the zinc-induced HIF-1 inhibition in tumor treatments? It is well known that hypoxia leads to chemoresistance which is a significant obstacle to successful cancer treatment [6]. One of the mechanisms of apoptosis resistance could be through expression of GLUT-1 or glycolytic enzymes, e.g., *Aldolase A/C* and hexokinases [41-43]. Besides these protective mechanisms that depend on glucose metabolism, alternative survival mechanisms that block apoptosis under hypoxia have been also implicated. These survival pathways may involve overexpression of anti-apoptotic proteins, e.g., Bcl-2 and inhibition or downregulation of pro-apoptotic proteins such as Bid or Bax [43]. Here, we found that zinc supplementation markedly reversed both the glycolytic pro-survival and anti-apoptotic pathways (see Table 1), leading to restoration of chemosensitivity under hypoxia. Thus, the ADR-induced gene expression, partially abrogated by the cobalt treatment, was restored by zinc supplementation almost to the original expression level of ADR (see Fig. 1A, Fig. 2), therefore giving a molecular answer to the initial question.

Recently, HIF-1 has been shown to antagonize the p53 apoptotic activity [44] and in particular we showed that this depends on HIF-1-induced HIPK2 downregulation [45]. A major determinant of a successful cancer therapy is the ability of cancer cells to activate apoptotic cell death, mainly through intact p53 function, and much experimental and pre-clinical effort is devoted to reactivation of inactive and/or mutant p53. Therefore, by assuming that a solid tumor presents intratumoral hypoxia, the zinc-induced HIF-1 inhibition should rescue the p53 function in tumors bearing wtp53 and HIPK2. As a proof of principle, we found here that one of the ubiquitine ligases involved in HIPK2 degradation under hypoxia, that is WSB-1 [35, 36], was up-regulated by cobalt but downregulated by zinc supplementation, strongly supporting the role of zinc in reactivating the HIPK2/p53 oncosuppressor axis under hypoxia. Another molecule important in p53 regulation is the MDM2 oncogene that downregulates both p53 and HIPK2 protein levels, strongly impairing tumor treatments [46]. Here, we found that MDM2 was one of the genes that were down-regulated by zinc (Table 4), supporting, at molecular level, the zinc inhibitory effect on MDM2 activity [17, 47].

Although much more effort must be taken to understand the physiological implications of the various pathways modified by hypoxia and/or cobalt and after zinc supplementation, our findings provide the molecular basis for the reversal of the hypoxia-induced changes of gene expression by zinc, which may have an effective anticancer outcome through a more efficient utilization of chemotherapy.

## MATERIALS AND METHODS

### Cell lines and treatments

RKO human colon carcinoma cells (wild-type p53) were maintained in RPMI-1640 (Life Technology-Invitrogen) supplemented with 10% heat-inactivated fetal bovine serum plus glutamine and antibiotics in humidified atmosphere with 5% CO_2_ at 37^0^ C. Subconfluent cells were seeded the day before the treatment. The cells were pre-treated for 16 hours with CoCl_2_ (200μM) and ZnCl_2_ (100 μM) before adding adryamicin (ADR) (1.5 μM) for additional 8 hours. The list of the different combination treatments used for the analyses is shown in Table 2, each treatment was prepared in duplicates.

### RNA extraction and reverse transcription (RT)-PCR analysis

After treatments, cells were harvested in TRIzol Reagent (Invitrogen) and total RNA was isolated following the manufacturer's instructions. The first strand cDNA and the semi-quantitative RT-PCRs were carried out essentially as described [17, 18]. Part of the cDNA was used to validate the system through analyses of gene transcription by RT-PCR by using genes specific oligonucleotides under conditions of linear amplification (data not shown). Total RNA was hybridized to Affymetrix HU-gene st1.0 microarrays.

### Data analysis

For normalization of the data from the 28,830 probesets of the arrays, Iter-Plier [48] algorithm was used on the .cel files, followed by modified Lowess correction algorithm [49] and log2 transformation. The mean intensity of each probe set in each pair of replicates was calculated, producing ‘averaged data' for nine different conditions. The histograms of these nine conditions are plotted in Supplementary Figure S1. Intensity dependent variance was estimated for each probe set, using the distribution of the differences between repeats of probe sets having similar mean intensity [50]. To look for differentially expressed probe sets between the different conditions, 17 comparisons were made (described in Supplementary Table S1), Each comparison was made between two corresponding pairs of replicates, using a filtering step which included probe sets that were above threshold (t=4) in both replicates, in at least one of the conditions. FDR of 5% was applied on all probe sets to control the number of false positives. An additional filter was imposed, requiring the fold change of the average gene expression to exceed 1.5. Unique gene representation of the data (known gene symbols only) was calculated for each gene symbol by averaging over all probe sets of that gene, and by averaging the replicates of each treatment.

To extract some biological meaning from differentially expressed genes, we looked at gene sets that share a common biological function such as cellular pathways. In order to do this we intersected the list of co-expressed genes with a subset of gene sets from the MsigDB [19] dataset, that belong to the positional, canonical pathways and GO biological process collections, retaining gene sets of minimal size of 8 and maximal size of 500 genes. Each gene set was assigned an enrichment score using the hyper geometric test; sets that passed FDR of 10% and included at least 5 genes were considered as significantly enriched.

## Supplementary Material


