# Supplementary material for: Genome-wide analysis discloses reversal of the hypoxia-induced changes of gene expression in colon cancer cells by zinc supplementation
Source: Oncotarget. 2011 Dec 24;2(12):1191–202. doi: 10.18632/oncotarget.395 (PMC3282077; doi:10.18632/oncotarget.395)
Supplement: Supplementary file 1 [file oncotarget-02-1191-s001.pdf]

Supplementary Material

Genome wide analyses discloses reversal of the hypoxia-induced changes of gene expression in cancer cells by zinc

Michal Sheffer[1], Amos J. Simon[2], Jasmine Jacob-Hirsch[2], Gideon Rechavi[2], Eytan Domany[1], David Givol[3] and Gabriella D'Orazi[4,5]

[1]Department of Physics of Complex Systems, Weizmann Institute of Science, Rehovot 76100, Israel;

[2]Department of Pediatric Hematology-Oncology, Chaim Sheba Medical Center and Sackler School of Medicine, Tel-Aviv University, Tel-Aviv, Israel;

[3]Department of Molecular Cell Biology; Weizmann Institute of Science, Rehovot 76100, Israel;

[4]Department of Experimental Oncology; Molecular Oncogenesis Laboratory; National Cancer Institute Regina Elena; Rome, Italy;

[5]Department of Oral Sciences, Nano and Biotechnology, University "G. d'Annunzio", Chieti, Italy.

Correspondence and requests for material should be addressed to: D.G. (david.givol@weizmann.ac.il) or to G.D.O. (gdorazi@unich.it).

Supplementary Table S1: Differential gene expression in each cell treatment. Cells were treated with different combinations of ADR (A), $ZnCl_2$ (Z) or $CoCl_2$ (C) and gene expression was compared between samples or untreated cells (0). The number of up- or down-regulated genes (by at least 1.5 fold change) is given for each comparison as described in Materials and Methods.

Comparison		Number of genes	
		**up-regulated**	**down-regulated**
1.	**A-0**	**2045**	**2369**
2.	**Z-0**	**37**	**35**
3.	**C-0**	**840**	**886**
4.	**AZ-A**	**77**	**39**
5.	**AZ-Z**	**2033**	**2206**
6.	**AC-A**	**1739**	**2105**
7.	**AC-C**	**514**	**643**
8.	**ZC-Z**	**34**	**17**
9.	**ZC-C**	**511**	**480**
10	**AZC-AZ**	**103**	**43**
11	**AZC-AC**	**2031**	**1566**
12	**AZC-ZC**	**2181**	**2579**
13	**AZ-0**	**1876**	**1997**
14	**AC-0**	**1378**	**2221**
15	**ZC-0**	**87**	**41**
16	**AZC-0**	**2168**	**2631**

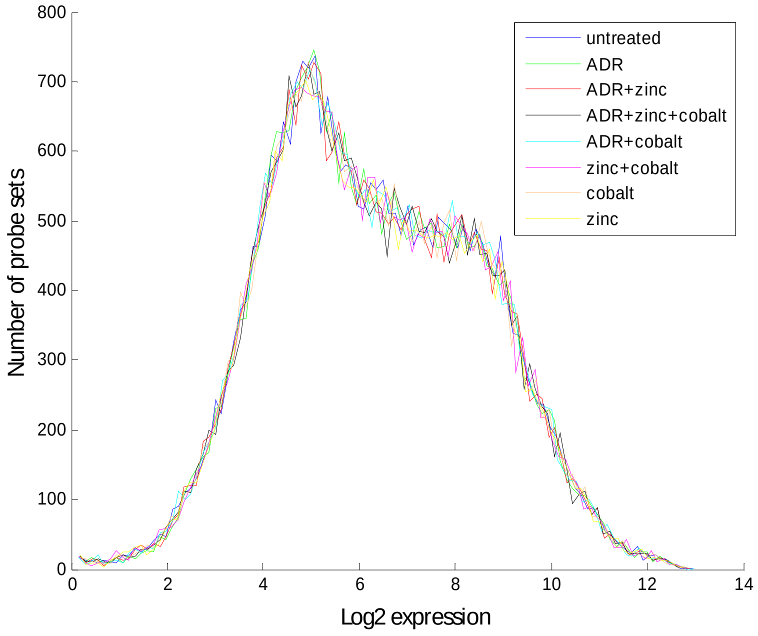

Supplementary Figure S1: Histogram of the samples. The different treatments are shown; each treatment is represented by the average log2 expression of its duplicates.
